# Ultrafast Cone-Beam Computed Tomography: A Comparative Study of Imaging Protocols during Image-Guided Therapy Procedure

**DOI:** 10.1155/2015/467850

**Published:** 2015-03-22

**Authors:** Jijo Paul, Annamma Chacko, Mohammad Farhang, Shahram Kamali, Mohsen Tavanania, Thomas Vogl, Bita Panahi

**Affiliations:** ^1^Diagnostic and Interventional Radiology, University Hospital, Goethe University Frankfurt, Theodor-Stern-Kai 7, 60590 Frankfurt, Germany; ^2^Division of Mathematics and Statistics, Dougherty System, Albany, GA 31705, USA

## Abstract

*Objective*. To evaluate two ultrafast cone-beam CT (UF-CBCT) imaging protocols with different acquisition and injection parameters regarding image quality and required contrast media during image-guided hepatic transarterial chemoembolization (TACE). *Methods*. In 80 patients (male: 46, female: 34; mean age: 56.8 years; range: 33–83) UF-CBCT was performed during TACE for intraprocedural guidance. Imaging was performed using two ultrafast CBCT acquisition protocols with different acquisition and injection parameters (imaging protocol 1: acquisition time 2.54 s, and contrast 6 mL with 3 s delay; imaging protocol 2: acquisition time 2.72 s, and contrast 7 mL with 6 s delay). Image evaluation was performed with both qualitative and quantitative methods. Contrast injection volume and dose parameters were compared using values from the literature. *Results*. Imaging protocol 2 provided significantly better (*P* < 0.05) image quality than protocol 1 at the cost of slightly higher contrast load and patient dose. Imaging protocol 1 provided good contrast perfusion but it mostly failed to delineate the tumors (*P* < 0.05). On the contrary, imaging protocol 2 showed excellent enhancement of hepatic parenchyma, tumor, and feeding vessels. *Conclusion*. Tumor delineation, visualization of hepatic parenchyma, and feeding vessels are clearly possible using imaging protocol 2 with ultrafast CBCT imaging. A reduction of required contrast volume and patient dose were achieved due to the ultrafast CBCT imaging.

## 1. Introduction

Cone-beam computed tomography (CBCT) is routinely used for many clinical applications in the fields of neurology, vascular, radiology, oncology, and cardiovascular interventions [1–9]. As an interventional oncology application, this system is routinely used for image-guiding purpose during transarterial chemoembolization (TACE) therapy procedures [10–12]. TACE guidelines recommend the use of contrast enhanced CBCT to delineate tumour and tumour feeding vessels [13, 14]. Many publications showed how intraprocedurally acquired CBCT images help to map out an adequate embolization strategy by visualizing the vessel tree that feeds hepatic tumors and metastases [8, 9, 14–17]. Modern angiographic systems are capable of acquiring cross-sectional CBCT image datasets during interventional procedures and improve the visualization of hepatic tumors as well as vascular anatomy [10–14]. However, CBCT implicates certain important limitations such as limited soft tissue resolution, limited field of view, and long acquisition times (typically 5 to 10 seconds in abdominal imaging) in general.

For contrast enhanced CBCT as performed during TACE procedures the long acquisition time also results in a long injection time as the contrast bolus has to be maintained during the whole time of the acquisition to provide a consistent filling of the imaged vessels and tumors. This means that the longer the acquisition time is the longer the contrast injection has to last and the more the contrast medium is required which can become a serious problem for patients with poor kidney function. The long acquisition time of image data is due to the limited maximum detector readout speed (30–60 f/s), limited maximum mechanical speed of CBCT system (40–60°/s), and number of projection images required for certain image quality. The present study intended to evaluate ultrafast CBCT (UF-CBCT) imaging protocols on a multiaxis robotic CBCT system regarding the capability to create image datasets suitable for guiding TACE procedures while at the same time save contrast media and radiation dose compared to values in the literature.

## 2. Material and Methods

### 2.1. Patient Characteristics

This is a prospective study conducted from October 2011 to June 2013 using 80 patients and the study protocol was accepted by institutional review board. The patient selection was randomly performed for each protocol without bias. The selection of the patients for UF-CBCT imaging was completely dependent on TACE inclusion and exclusion criteria, based on previous publication [12]. Inclusion criteria for the TACE therapy were as follows: confirmation of at least a single tumor in the liver parenchyma, unresectable metastatic tumor(s), contraindication to surgery, and tumor(s) not responsive to radiotherapy or chemotherapy. Exclusion criteria were as follows: existence of extrahepatic tumor(s), poor patient performance status, poor hepatic function, renal failure, contraindication to angiography, respiratory or cardiovascular failure, obstructive jaundice, portal vein thrombosis or occlusion, and recently received radiotherapy (in last two months). MRI is considered to be gold standard imaging modality for detection of tumors present in the hepatic parenchyma. Pretreatment T1 weighted unenhanced/contrast enhanced and T2 weighted unenhanced/contrast enhanced magnetic resonance images (MRI) were acquired for all patients to assess the hepatic tumor details such as size, shape, number, and position.

### 2.2. Cone-Beam CT Imaging

We used a multiaxis robotic CBCT system (Artis zeego, Siemens Healthcare, Forchheim Germany) to conduct patient examinations during TACE therapy. This UF-CBCT system offers the possibility to rotate the tube-detector system around the patient with a maximum speed of up to 100°/sec. The system is equipped with a latest generation 30 × 40 cm flat panel detector made of amorphous silicon with cesium iodide scintillator (aSi:CsI). During UF-CBCT acquisition the system acquires projection images on a 200° circular trajectory with a constant angular frame increment (AI); that is, each AI degree the system acquired an image. This means that the image acquisition frame rate differs between the acceleration phase, the phase with constant speed, and the deceleration phase of the CBCT.

The correlation between UF-CBCT rotation speed (*v*_cbct_), readout speed (*v*_ro_), and angular frame increment (AI) is as follows:

(1) AI(/°image)=vcbct(/°s)  vro(image/s).

With a given angular increment for a UF-CBCT acquisition the maximum readout speed of the detector may limit the maximum CBCT rotation speed.

### 2.3. Data Acquisition Protocols

In this study we evaluated two UF-CBCT imaging protocols, each consisting of a CBCT acquisition protocol and a contrast injection protocol (Table 1). The angular increment of acquisition protocol 1 (Table 1) was set to 1.5° per image, resulting in 133 images on the 200° circular trajectory. The angular increment of acquisition protocol 2 was set to 1.2° per image, resulting in 166 images on the 200° circular trajectory.

For the UF-CBCT protocols the CBCT system had to be positioned in a head side position so that the acquisition is performed in a “propeller-like” mode where only one axis of the multiaxis robotic UF-CBCT system is moved. The 200° acquisition trajectory reaches from right-anterior-oblique (RAO) 170° to left-anterior-oblique (LAO) 30°. During TACE, the patients were positioned head-first in supine position on table top and their arms were positioned above the head during data acquisition. Furthermore, UF-CBCT image data was acquired for all patients on expiration condition. The UF-CBCT acquisition was performed with a 0.36 *μ*Gy/image detector entrance dose setting. The tube voltage is preset to 90 kV but is modulated together with the tube current during the rotational run to keep the detector entrance dose constant.

### 2.4. Image Data Reconstruction

From the projection images a 3D dataset with isotropic voxels of 0.5 mm is automatically reconstructed on the connected workstation (syngo XWP, Siemens Healthcare, Forchheim, Germany) using a filtered back-projection (Feldkamp) algorithm. This 3D dataset is then loaded into the software application syngo InSpace, which allows the user to visualize the dataset in different rendering modes like multiplanar reformatted (MPR), maximum intensity projection (MIP), or volume rendered (VRT) images.

### 2.5. Contrast Injection Protocol

A UF-CBCT acquisition with contrast material injection (Visipaque 320 from GE Healthcare Braunschweig, Germany) was performed for all examined patients. The injection was performed into the right or left hepatic artery using a coaxial microcatheter (2.7F/2.4F × 150 cm; Trevo Pro 18 microcatheter, Concentric medical, CA, USA). A road mapping of the target tumor is possible using contrast material injection as described by Wallace et al. [18]. Contrast injection protocol 1 was used in conjunction with acquisition protocol 1 (Table 1) on forty patients (22 male, 18 female; mean age: 56.3 years; range: 33–73 years) during imaging (imaging protocol 1). Contrast injection protocol 2 was used in conjunction with acquisition protocol 2 on another forty patients (24 male, 16 female; mean age: 57.3 years; range 43–83 years) during TACE (imaging protocol 2). Complete information regarding the parameters used for contrast material injection during both image acquisitions is provided in Table 1.

### 2.6. Image Analysis

After the examination of patients using UF-CBCT, the images were evaluated by three radiologists with 4, 6, and 20 years of experience in abdominal imaging. They used a scoring system to analyze image data qualitatively based on tumor delineation, vascular contrast material perfusion, and appearance of artifacts on the cross-sectional images (Table 2). Readers were blinded regarding imaging protocols and associate parameters used for the study but they were informed the images were generated during TACE therapy procedure. The number of tumors which appeared on image data was assessed by the same radiologists. The hepatic tumors were classified as three categories during analysis based on its enhancement characteristics: hypoenhanced tumor, heterogeneously enhanced tumor, and homogenously enhanced tumor (Table 3). Moreover, quantitative image quality parameters such as Hounsfield unit (HU), image noise, signal-to-noise ratio (SNR = HU/noise), and tumor-to-liver contrast (TLC = HU_tumor_ − HU_liver_) were also determined using the acquired UF-CBCT image datasets. Quantitative measurements were performed by the same radiologists according to the previous publications [2, 12, 14]. HU was measured using a circular region of interest (ROI) placed on the hepatic parenchyma away from the tumor for normal parenchymal measurements and on the tumor for tumor HU measurements. Diameter of the circular ROI used for measurement of HU was 2 cm; however, this mentioned diameter may change according to the size of the tumor during tumor HU measurements. Two ROI measurements were performed using adjacent CBCT slices and average value was taken into account for calculating mean HU values. A standard deviation of pixel values in the ROI circle was considered as image noise (HU). All measurements were performed using a dedicated syngo X-Leonardo workstation from Siemens Healthcare, Forchheim, Germany.

### 2.7. Patient Dose Analysis

Two radiation dose parameters, dose area product (DAP) and patient entrance dose (PED), were obtained during exposure from the patient examination protocol generated on the UF-CBCT system [10]. Calculated values were compared using the data available in the literature.

### 2.8. Statistical Analysis

Results of the present study are displayed as means ± standard deviation and range for continuous variables. The statistical analyses were performed using computer based BiAS software (BiAS for Windows, Epsilon 2008, version 8.4.2). A *P* value less than or equal to 0.05 is considered as statistically significant results. The normality of data distribution was examined using Kolmogorov-Smirnov-Lilliefors test. Qualitative image quality assessment comparisons were performed using Wilcoxon rank sum test; furthermore, interobserver comparisons were performed using Cohen's Kappa during qualitative analysis. Kappa agreement was considered *k* < 0 (less than chance agreement), *k* = 0.01–0.20 (slight agreement), *k* = 0.21–0.40 (fair agreement), *k* = 0.41–0.60 (moderate agreement), *k* = 0.61–0.80 (substantial agreement), and *k* = 0.81–0.99 (almost perfect agreement). Regarding comparison of the quantitative results, paired Student's *t*-test was used to test the significance between data categories. A gold standard pretreatment MR-image data was used as standard of reference for statistical analysis during tumor detection. Sensitivity and predictive values were determined in relation to the detectability of hepatic tumors.

## 3. Result

**Figure 1:**
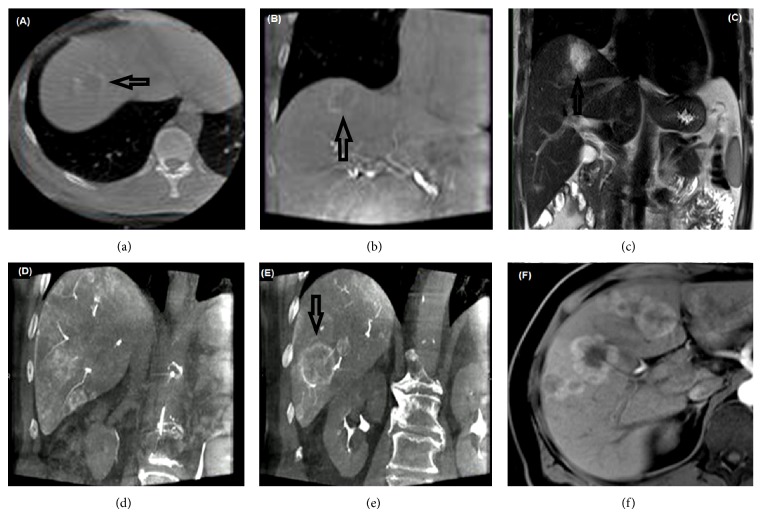
Frames (a) and (b) represented the images obtained from a 62-year-old patient during TACE therapy, generated using ultrafast cone-beam CT with imaging protocol 1. Pretreatment magnetic resonance cross-sectional images ((c) and (f)) show a clear view of embedded tumor in the hepatic parenchyma. Hepatic tumor detection was insufficient using imaging protocol 1 data (tumor indicated using black arrow) compared to imaging protocol 2 due to a reduction of contrast material in the tumors. Coronal reconstructed images ((d) and (e)) were acquired using imaging protocol 2 during a 60-year-old patient TACE examination. The images show excellent tumor(s), feeding vessels, and hepatic parenchymal visualization; furthermore, notice the strong contrast material enhancement of the tumors with little or no artifacts.

Details of the patient hepatic tumor characteristics obtained using gold standard MRI are displayed in Table 3 and tumor characteristics determined using images of both UF-CBCT imaging protocols are provided in Table 4. The evaluated imaging protocol 2 produced an excellent tumor delineation, contrast perfusion, and parenchymal visualization (Figure 1; Table 5) with adequate enhancement due to the use of proper bolus timing, mixing ratio, and X-ray delay time during ultrafast imaging. However, using imaging protocol 1 the identification of hepatic tumors was difficult because of reduced contrast material (Figure 1) as a result of improper delay time for ultrafast imaging. Qualitative analysis showed large difference of image quality between the two imaging protocols (Table 5). Image quality was significantly higher (all *P* < 0.05) in imaging protocol 2 compared to imaging protocol 1 (Table 5; Figure 1). Interreader agreement performed using Kappa during qualitative analysis showed almost perfect agreement (*K* = 0.832–0.947).

Quantitative image quality analysis also showed similar results to qualitative analysis (Table 6). Based on quantitative analysis HU was significantly higher (all *P* < 0.05) in imaging protocol 2 compared to 1 (Table 6). The calculated SNR values were significantly higher (all *P* < 0.05) in protocol 2 image data compared to the other protocol evaluated. The TLC computation to determine quantitative tumor delineation showed significantly higher (all *P* < 0.05) results for protocol 2 compared to 1 (Table 6).

The volume of iodine injected into the patients during UF-CBCT was 1920 mg and 2240 mg, respectively, for injection protocols 1 and 2 during contrast material injection. In [14], authors performed three conventional CBCT patient examinations during TACE therapy. They used 4800 mg, 6400 mg, and 8000 mg of iodine in the injected volume of contrast material during hepatic CBCT imaging. In [19] authors used 4625 mg and [20] used 9000 mg of iodine in the injected volume of contrast for patient examinations. UF-CBCT imaging protocol 2 used a marked reduction of iodine volume compared to [14] by 53%, 65%, and 72% and [19] by 51.5%. Moreover, a significant reduction of iodine (75%) was observed during UF-CBCT protocol 2 compared with [20]. Tumor detection sensitivity and predictive values were calculated using the image data obtained from UF-CBCT and MRI. Determined sensitivity showed a remarkable increase in UF-CBCT imaging protocol 2 data compared to 1 (Table 7).

Mean PED estimated for UF-CBCT imaging protocols 1 and 2 were 77.5 ± 12.2 mGy (69–91) and 81.6 ± 12.8 mGy (72–104), respectively, while the mean DAP obtained for imaging protocols 1 and 2 were 18.37 ± 4.4 Gy·cm² (11–26) and 22.55 ± 4.9 Gy·cm² (15–29), respectively. Reference [12] showed a mean PED of 111.8 ± 12.8 mGy (101–128) and DAP value of 29.2 ± 8 Gy·cm² (21–36) during TACE therapy using 5s tube-detector rotation. However, [2] displayed a mean PED of 124.4 ± 19.5 mGy (109.5–136) and DAP of 32.3 ± 5 Gy·cm² (26–37) during TACE using 5s protocol. UF-CBCT imaging protocol 2 showed a reduction of radiation dose by 22.8% (DAP)/27% (PED) from [12] and 30.3% (DAP)/34.4% (PED) from [2], respectively, during comparison.

## 4. Discussion

Long patient breath hold is necessary for conventional CBCT image data acquisition to avoid motion artifacts and thus obtain reasonable image quality. A continuous contrast material injection should be maintained in entire duration during contrast enhanced CBCT data acquisition. These two conditions directly affect image quality as well as contrast material load in the patients. The high rotation speed of the tube-detector system and shorter acquisition time of the UF-CBCT make it easier for patients to comply with the breath-hold requirements and thus reduce the occurrence of motion artifacts, which produce good image quality. Ultrafast CBCT imaging bears the potential to reduce volume of contrast material (up to 6 and 7 mL) required for contrast enhanced CBCT during TACE therapy of patients due to a reduction of imaging time and appropriate contrast protocol used for imaging. The volume of iodine in the contrast material injected during CBCT imaging as regards TACE is significantly different in various published materials [14, 19–21] ranging from 4000 to 9000 mg. In the present study, we used only 1920 mg and 2240 mg of iodine in the injected volume of contrast using injection protocols 1 and 2, respectively. Since many patients with malignant liver tumors require multiple embolization sessions, the evaluated UF-CBCT imaging protocols could help to reduce the “life-time” volume of contrast material significantly [2].

We obtained a reduced radiation dose on patients due to ultrafast imaging time compared to published data [2, 12]. The evaluated UF-CBCT imaging protocols generate a reduced number of images (from 248 to 133 during imaging protocol 1 and 166 during imaging protocol 2) with the same per frame system dose compared to imaging protocols evaluated in previous publications [2, 12]. At least imaging protocol 2 proved to produce sufficient image quality for TACE guidance with reduced patient dose.

Injection protocols and delay time are highly influencing parameters on the enhancement of hepatic parenchyma and tumors. Imaging protocol 1 produced an insufficient visualization of hepatic tumors/metastasis. This is due to the short X-ray delay time of 3 seconds, which prevented proper tumor enhancement before the UF-CBCT data acquisition. In imaging protocol 2, the X-ray delay was extended to 6 seconds which allowed the further perfusion of the tumor feeding vessels with contrast material. To keep the contrast load for the patient at a reasonable level the contrast dilution was increased from a mixing level of 1 : 2 (contrast/saline) in imaging protocol 1 to 1 : 3 in imaging protocol 2. Despite the higher contrast/water dilution compared to imaging protocol 1, imaging protocol 2 produced excellent tumor enhancement with the possibility of good tumor delineation during UF-CBCT imaging. The reasons are the higher delay time and the higher number of frames acquired. To obtain reasonable image quality for CBCT image data a certain number of projection images (frames) are required [10]. Ideally a CBCT system should be able to acquire certain desired number of projection images in a very short time and the tube-detector system should be able to rotate at a very high speed while these projection images are acquired. In this study we utilized high frame rate and a robotic CBCT system with ultrafast rotation capabilities resulting in a very short data acquisition time for UF-CBCT acquisition. During acquisition protocol 1 imaging, the UF-CBCT is rotating with its highest possible speed, that is, 100°/sec; based on formula 1 a frame rate of 67 images per second is required to read out an image every 1.5°. The system accelerates to the maximum speed in approximately 1 second and drives at maximum speed for about 1 sec before it decelerates in 0.5 sec to a stop again. Hence, the total acquisition time of protocol 1 is 2.54 seconds. In protocol 2, the maximum readout speed of the available detector is limited to 74 frames per second; the maximum CBCT speed had to be limited to 88.8°/s (see formula 1). With an acceleration phase of ~0.8 seconds, a phase of maximum speed of ~1.5 seconds, and a deceleration phase of 0.4 seconds the total acquisition time of protocol 2 sums up to 2.72 seconds.

In the present study we used an ultrafast robotic CBCT imaging system and protocols with less than 3 seconds image acquisition time acquiring image data during TACE. Imaging protocol 2 provided an excellent visualization of tumor(s) and feeding vasculature as well as hepatic parenchyma due to adequate bolus timing, mixing ratio X-ray delay time, and higher number of acquired frames compared to imaging protocol 1. Ultrafast image acquisitions reduce contrast material injection volume to patients during TACE examination using UF-CBCT. Furthermore, UF-CBCT imaging achieved a reduction of radiation dose due to reduction of total number of acquired frames during imaging. The reduction of imaging time helps to prevent the appearance of motion artifacts which was previously reported as a problem with longer CBCT acquisitions. Based on image quality results of imaging protocol 2 we recommend that this imaging protocol should be used for UF-CBCT image acquisitions during patient imaging.

## Figures and Tables

**Table 1:** Described specifications of the clinical imaging parameters and contrast material injection protocol used for the ultrafast cone-beam CT imaging of patients during transarterial chemoembolization treatment.

Imaging parameters	Acquisition protocol 1	Acquisition protocol 2
Set kilo-voltage	90 kV	90 kV
Number of images	133	166
Angular increment (°/ima)	1.5	1.2
Maximum CBCT speed (°/s)	100	88.8
Maximum readout speed (ima/s)	67	74
Total acquisition time (s)	2.54	2.72
Detector entrance dose (uGy/frame)	0.36	0.36

Contrast injection parameters	Injection protocol 1	Injection protocol 2

Contrast volume (mL)	6	7
Iodine/mL	320	320
Saline (mL)	12	20
Flow rate (mL/s)	3	3
X-ray delay (s)	3	6
Injection duration (s)	6	9

**Table 2:** Classification of grading score based on tumor delineation, vascular contrast material perfusion, and appearance of the artifacts.

Grading point score	Tumor delineation, vascular contrast material perfusion, and appearance of artifacts.
	Description
1	Not suitable for diagnosis (nondiagnostic image data)
2	Suboptimal contrast perfusion and tumor delineation, strong appearance of artifacts
3	Less than standard contrast perfusion and tumor delineation with hazy appearance of artifacts
4	Standard contrast perfusion and tumor delineation with hazy appearance of artifacts
5	Vascular contrast material perfusion and tumor delineation are higher than necessary with little or no artifacts

**Table 3:** Details of patient tumor characteristics determined using gold standard MR-image data. The determined tumor characteristics using MRI displayed separately for UF-CBCT imaging protocols 1 (P1) and 2 (P2) patient groups.

Type of hepatic tumor	Tumor involvement	Hepatic tumor groups	Number of patients		Number of tumors		Mean dimension in cm (*l* × *b*)	
	(P1/P2)		P1	P2	P1	P2	P1	P2
Hypoenhanced tumor	Right lobe: 21/21	Metastasis from:						
	Left lobe: 13/14	Thyroid carcinoma	5	6	16	20	4.4 × 3.9	4 × 3.8
	Caudate lobe: 2/5	Colorectal carcinoma	6	7	25	23	4.8 × 4.7	4.5 × 4.4
	Quadrate lobe: 5/3							

Heterogeneously enhanced tumor	Right lobe: 21/24	Cholangiocarcinoma	5	4	17	15	4 × 3.8	3.8 × 3.6
	Left lobe: 15/12	Metastasis from:						
	Caudate lobe: 7/3	Colorectal carcinoma	4	7	12	12	4.3 × 3.7	4.5 × 4
	Quadrate lobe: 1/2	Breast carcinoma	4	3	15	14	4.8 × 4.3	4.3 × 3.9

Homogenously enhanced tumor	Right lobe: 26/22	Hepatocell. carcinoma	7	5	28	20	4.1 × 3.6	4 × 3.6
	Left lobe: 15/14	Cholangiocarcinoma	5	5	9	13	4.9 × 4.2	4.2 × 3.8
	Caudate lobe: 2/4	Metastasis from:						
	Quadrate lobe: 3/5	Colorectal carcinoma	4	3	9	12	4.7 × 4.4	4.5 × 3.8

**Table 4:** Details of the patient tumor characteristics obtained using ultrafast CBCT image data (P1: imaging protocol 1; P2: imaging protocol 2).

Type of hepatic tumor	Tumor involvement	Hepatic tumor groups	Number of patients		Number of tumors		Mean dimension in cm (*l* × *b*)	
	(P1/P2)		P1	P2	P1	P2	P1	P2
Hypoenhanced tumor	Right lobe: 16/19	Metastasis from:						
	Left lobe: 10/13	Thyroid carcinoma	5	6	11	19	3.9 × 3.4	3.8 × 3.5
	Caudate lobe: 0/5	Colorectal carcinoma	6	7	17	21	4.5 × 4.2	4.3 × 4.1
	Quadrate lobe: 2/3							

Heterogeneously enhanced tumor	Right lobe: 18/21	Cholangiocarcinoma	5	4	13	13	3.5 × 3.4	3.6 × 3.4
	Left lobe: 11/11	Metastasis from:						
	Caudate lobe: 4/3	Colorectal carcinoma	4	7	9	11	3.8 × 3.2	4.2 × 3.7
	Quadrate lobe: 0/2	Breast carcinoma	4	3	11	13	4.2 × 3.7	4 × 3.7

Homogenously enhanced tumor	Right lobe: 19/21	Hepatocell. carcinoma	7	5	21	19	3.6 × 3.1	3.7 × 3.3
	Left lobe: 11/13	Cholangiocarcinoma	5	5	6	12	4.6 × 3.6	3.9 × 3.5
	Caudate lobe: 2/4	Metastasis from:						
	Quadrate lobe: 2/5	Colorectal carcinoma	4	3	7	12	4 × 3.8	4.2 × 3.5

**Table 5:** Displayed image quality qualitative analysis scores (mean ± standard deviation and range) obtained from the readers using both ultrafast CBCT patient imaging protocols.

Imaging protocol	Tumor classification:		
	Hypoenhanced tumor (A)	Heterogeneously enhanced tumor (B)	Homogenously enhanced tumor (C)
Protocol 1	2.9 ± 0.4 (2.6–3.1)	3 ± 0.4 (2.6–3.25)	3.1 ± 0.5 (2.7–3.4)
Protocol 2	4 ± 0.6 (3.7–4.2)	4.3 ± 0.4 (4–4.5)	4.6 ± 0.3 (4.3–4.8)

*P* value	0.0001	0.0001	0.0001

**Table 6:** Quantitative image quality parameters and tumor delineation obtained from ultrafast CBCT image data with contrast material injection for both examined protocols. Furthermore, TLC represents tumor-to-liver contrast.

Image quality parameter	Measurement locations			
	Normal hepatic parenchyma	Hypoenhanced tumor (A)	Heterogeneously enhanced tumor (B)	Homogenously enhanced tumor (C)
Protocol 1				
Hounsfield unit (HU)	54 ± 13 (41–68)	25 ± 8 (14–37)	63 ± 15 (47–77)	151 ± 17 (119–196)
Image noise (HU)	42 ± 11 (37–49)	52 ± 18 (39–59)	62 ± 17 (51–73)	83 ± 22 (69–99)
Signal-to-noise ratio (SNR)	1.3 (1.15–1.4)	0.48 (0.35–0.58)	1 (0.98–1.1)	1.8 (1.7–1.93)
Tumor-to-liver contrast (TLC)	—	−29 (−18–−39)	9 (4–14)	97 (91–113)

Protocol 2				
Hounsfield unit (HU)	83 ± 14 (69–95)	10 ± 3 (6–15)	115 ± 20 (74–147)	217 ± 21 (169–244)
Image noise (HU)	29 ± 9 (25–35)	17 ± 7 (13–23)	44 ± 15 (32–52)	60 ± 19 (49–66)
Signal-to-noise ratio (SNR)	2.85 (2.75–3)	0.7 (0.4–0.8)	2.6 (2.3–2.8)	3.6 (3.5–3.7)
Tumor-to-liver contrast (TLC)	—	−73 (−59–−89)	32 (21–41)	134 (119–162)

*P* value (protocol 1 versus protocol 2):	0.0001	0.0001	0.0001	0.0001

**Table 7:** Displayed sensitivity and predictive values, calculated using UF-CBCT imaging protocols 1 and 2 and magnetic resonance image data.

Type of tumor	Interpreted as tumor on		True positive	False negative	False positive	True negative	Sensitivity (%)	Specificity (%)	Positive predictive value (%)	Negative predictive value (%)
	MRI	UF-CBCT protocol 1								
Hypoenhanced tumors	41	28	23	18	5	0	56	0	82	0
Heterogeneously enhanced tumors	44	33	27	17	6	0	61.3	0	81.8	0
Homogeneously enhanced tumors	46	34	29	17	5	0	63	0	85.3	0

	MRI	UF-CBCT protocol 2								

Hypoenhanced tumors	43	40	36	7	4	0	83.7	0	90	0
Heterogeneously enhanced tumors	41	37	34	7	3	0	82.9	0	91.9	0
Homogeneously enhanced tumors	45	43	41	4	2	0	91	0	95.3	0

## References

[1] Orth R. C., Wallace M. J., Kuo M. D. (2008). C-arm cone-beam CT: general principles and technical considerations for use in interventional radiology. *Journal of Vascular and Interventional Radiology*.

[2] Paul J., Mbalisike E. C., Vogl T. J. (2013). Radiation dose to procedural personnel and patients from an X-ray volume imaging system. *European Radiology*.

[3] Jeon U. B., Lee J. W., Choo K. S. (2009). Iodized oil uptake assessment with cone-beam CT in chemoembolization of small hepatocellular carcinomas. *World Journal of Gastroenterology*.

[4] Iwazawa J., Ohue S., Hashimoto N., Abe H., Hamuro M., Mitani T. (2010). Detection of hepatocellular carcinoma: comparison of angiographic C-arm CT and MDCT. *American Journal of Roentgenology*.

[5] Morimoto M., Numata K., Kondo M. (2010). C-arm cone beam CT for hepatic tumor ablation under real-time 3D imaging. *The American Journal of Roentgenology*.

[6] Kempfert J., Noettling A., John M., Rastan A., Mohr F. W., Walther T. (2011). Automatically segmented DynaCT: enhanced imaging during transcatheter aortic valve implantation. *Journal of the American College of Cardiology*.

[7] Dijkstra M. L., Eagleton M. J., Greenberg R. K., Mastracci T., Hernandez A. (2011). Intraoperative C-arm cone-beam computed tomography in fenestrated/branched aortic endografting. *Journal of Vascular Surgery*.

[8] Schwartz J. G., Neubauer A. M., Fagan T. E., Noordhoek N. J., Grass M., Carroll J. D. (2011). Potential role of three-dimensional rotational angiography and C-arm CT for valvular repair and implantation. *International Journal of Cardiovascular Imaging*.

[9] Dörfler A., Struffert T., Engelhorn T., Richter C. (2008). Rotational flat-panel computed tomography in diagnostic and interventional neuroradiology. *RoFo*.

[10] Paul J., Jacobi V., Farhang M., Bazrafshan B., Vogl T. J., Mbalisike E. C. (2013). Radiation dose and image quality of X-ray volume imaging systems: cone-beam computed tomography, digital subtraction angiography and digital fluoroscopy. *European Radiology*.

[11] Miyayama S., Yamashiro M., Okuda M. (2011). Detection of corona enhancement of hypervascular hepatocellular carcinoma by C-arm dual-phase cone-beam CT during hepatic arteriography. *CardioVascular and Interventional Radiology*.

[12] Paul J., Vogl T. J., Mbalisike E. C. (2012). Radiation dose and image quality evaluation relative to different contrast media using cone-beam CT. *Imaging in Medicine*.

[13] Lencioni R., de Baere T., Burrel M. (2012). Transcatheter treatment of hepatocellular carcinoma with doxorubicin-loaded dc bead (DEBDOX): technical recommendations. *CardioVascular and Interventional Radiology*.

[14] Koelblinger C., Schima W., Berger-Kulemann V. (2013). C-arm CT during hepatic arteriography tumour-to-liver contrast: intraindividual comparison of three different contrast media application protocols. *European Radiology*.

[15] Sun J.-H., Wang L.-G., Bao H.-W. (2010). Usefulness of C-arm angiographic computed tomography for detecting iodized oil retention during transcatheter arterial chemoembolization of hepatocellular carcinoma. *Journal of International Medical Research*.

[16] Lin M., Loffroy R., Noordhoek N. (2011). Evaluating tumors in transcatheter arterial chemoembolization (TACE) using dual-phase cone-beam CT. *Minimally Invasive Therapy and Allied Technologies*.

[17] Loffroy R., Lin M., Rao P. (2012). Comparing the detectability of hepatocellular carcinoma by C-arm dual-phase cone-beam computed tomography during hepatic arteriography with conventional contrast-enhanced magnetic resonance imaging. *CardioVascular and Interventional Radiology*.

[18] Wallace M. J., Kuo M. D., Glaiberman C., Binkert C. A., Orth R. C., Soulez G. (2009). Three-dimensional C-arm Cone-beam CT: applications in the Interventional Suite. *Journal of Vascular and Interventional Radiology*.

[19] Miyayama S., Yamashiro M., Okuda M. (2009). Usefulness of cone-beam computed tomography during ultraselective transcatheter arterial chemoembolization for small hepatocellular carcinomas that cannot be demonstrated on angiography. *CardioVascular and Interventional Radiology*.

[20] Higashihara H., Osuga K., Onishi H., Nakamoto A., Tsuboyama T., Maeda N. (2012). Diagnostic accuracy of C-arm CT during selective transcatheter angiography for hepatocellular carcinoma: comparison with intravenous contrast-enhanced, biphasic, dynamic MDCT. *European Radiology*.

[21] Iwazawa J., Ohue S., Mitani T. (2009). Identifying feeding arteries during TACE of hepatic tumors: comparison of C-Arm CT and digital subtraction angiography. *American Journal of Roentgenology*.

